# Survodutide acts through circumventricular organs in the brain and activates neuronal regions associated with appetite regulation

**DOI:** 10.1016/j.molmet.2026.102326

**Published:** 2026-02-02

**Authors:** Tina Zimmermann, Katherin Bleymehl, Peter Haebel, Johanna Perens, Urmas Roostalu, Jacob Hecksher-Sørensen, Jonas Doerr, Sebastian Jarosch, Daniel Lam, Holger Klein, Anton Pekcec, Samar N. Chehimi, Richard C. Crist, Benjamin C. Reiner, Matthew R. Hayes, Robert Augustin

**Affiliations:** 1 Boehringer Ingelheim Pharma GmbH & Co. KG, Birkendorfer Str. 65, Biberach an der Riβ, 88400, Germany; 2 Gubra, Hørsholm, Denmark; 3 University of Pennsylvania Perelman School of Medicine, Philadelphia, USA

**Keywords:** Survodutide, Obesity, Glucagon, Food intake, Circumventricular organs, Brain

## Abstract

Survodutide is a novel GCG/GLP-1 receptor (GCGR/GLP-1R) dual agonist in clinical development for people with obesity and people with metabolic dysfunction-associated steatohepatitis (MASH). Preclinically, survodutide demonstrated body weight lowering efficacy through decreased energy intake and increased energy expenditure. Here, we investigated the central site of action of survodutide and provide further insights into its mechanism of action in reducing body weight. We assessed *GCGR* and *GLP1R* expression in human and mouse circumventricular organs (CVOS) and showed for the first time that *GCGR* is barely detectable in area postrema (AP) and arcuate nucleus of the hypothalamus (ARH) at the single cell level. In contrast, *GLP1R* is expressed in these tissues. Using a fluorophore labeled survodutide to visualize sites of action in the mouse brain, survodutide was observed to directly access the CVOs and adjacent hypothalamic and hindbrain nuclei, without evidence of uniformly crossing the blood–brain-barrier. In addition, c-Fos labeling showed that multiple nuclei associated with the control of food intake were activated by survodutide. Consistent with the hypothesis that the intake suppressive effects of survodutide are GLP-1R dependent, a long-acting GCGR agonist did not induce neuronal activation in satiety-mediating regions, nor reduced food intake but showed reduction in body weight. These data further support the dual mode of action of survodutide and its potential to provide clinical benefit for people with obesity and/or MASH.

## Highlights

• Survodutide activates GLP-1R–expressing satiety nuclei, suppressing food intake.
• GCGR expression is minimal in AP and ARH, indicating limited central glucagon action.
• Survodutide accesses CVOs and adjacent nuclei without uniform BBB penetration.
• GCGR agonist did not activate satiety nuclei or suppress intake, despite weight loss.

## 1 Introduction

Survodutide is a novel glucagon/glucagon-like peptide-1 receptor (GCGR/GLP-1R) dual agonist currently in clinical development for people living with obesity or metabolic dysfunction-associated steatohepatitis (MASH). In Phase 2 studies survodutide demonstrated clinically relevant body weight lowering of up to 19% [1] and showed significant improvements in MASH resolution and fibrosis in people with MASH [2]. The mechanism of action of survodutide to lower body weight is a consequence of increases in energy expenditure by activation of the GCGR in the liver [3] and a reduction in energy intake by GLP-1R agonism in the brain. In mice, activating GCGR on top of GLP-1R resulted in greater bodyweight reductions compared to a selective GLP-1R agonist, semaglutide [4]. The peptide hormone oxyntomodulin, an endogenous, weak GLP-1R and glucagon receptor (GCGR) dual agonist, has been shown to increase cFos expression and reduce food intake, potentially via GLP-1R. However, only intracerebroventricular administration of oxyntomodulin reduced food intake, and not intraperitoneal administration [5], which might be attributed to the short half-life of oxyntomodulin and its lower potency [4] and therefore efficacy. In contrast to oxyntomodulin, survodutide is a highly potent dual GLP-1R and GCGR agonist (with 8-fold higher potency for the GLP-1R) with a prolonged half-life.

GCGR expression has been identified in the liver, kidney, and pancreatic islets [[6], [7], [8]], specifically on β-cells [[6], [7], [8]]. This localization underpins glucagon's role as a paracrine modulator of insulin secretion. Early studies by Samols and Marks demonstrated that intra-islet glucagon potently stimulates insulin release under physiological glucose conditions [[9], [10], [11], [12]]. Recent functional studies using β-cell–specific GCGR knockout models further established that glucagon enhances insulin secretion via GCGR-mediated cAMP signaling [13].

Within the central nervous system (CNS) limited reports exist on GCGR expression [6,14]. Binding sites in the rat brain for GCG have been reported at low levels in the hypothalamus, olfactory regions, hippocampus, anterior pituitary, amygdala, medulla, and thalamus [15,16]. In contrast to the limited literature on central GCGR expression, GLP-1Rs are well documented to be expressed throughout the rodent and nonhuman primate brains, including expression in many nuclei and neural substrates known to be involved in the control of food intake [17]. GLP-1R is detected in substantia nigra (SNC), ventral tegmental area (VTA), amygdala, nucleus accumbens (ACB), hippocampus, several regions of the hypothalamus (paraventricular nucleus of the hypothalamus (PVH), ARH), and a multitude of caudal brainstem nuclei [18,19]. In recent single cell (sc)/single nucleus (sn) RNA-seq atlases covering the hypothalamus of the mouse and the human brain [[20], [21], [22]], *GLP1R* expression is detected in small populations of neurons, while *GCGR* is not appreciably expressed. However, the precise expression of GCGR and GLP-1R in human peptide accessible CVOS (AP and ARH) is not known. In the present study, we provide a detailed transcriptomic map of these receptors in human AP and ARH. It should be noted, however, that functional target engagement by survodutide was assessed exclusively in mouse models, and not in human tissue.

Half-life extended, fluorescently labeled GLP-1R agonists liraglutide and semaglutide exhibit limited deep brain access following peripheral administration and activate neurons in the hypothalamus and the hindbrain that are involved in food intake control [23,24]. While initial studies primarily demonstrated regional labeling, Ast et al. [25] achieved cell anatomical and dendritic resolution, providing detailed insights into the localization of GLP-1R agonist binding and its spatial relationship to endogenous proglucagon-expressing neurons.

The putative mechanism by which glucagon (GCG) reduces body weight has been suggested to be increasing energy expenditure and reducing in energy intake. Specifically, a half-life extended long acting GCG has been assessed previously for its energy expenditure increasing component [26]. However, the effects on food intake have not been explored. Although with much lower potency, the endogenous peptide GCG also activates the GLP-1R [4,[27], [28], [29]]. Therefore, effects of GCG on food intake [30] might be mediated via the GLP-1R when investigated at suprapharmacological doses using unspecific tools or i.v. infusions of glucagon. The overarching aims of the present study are to investigate potential CNS sites of action for survodutide and to understand the putative impact of GLP-1R and GCGR in nuclei of relevance to energy balance control.

## 2 Results

### 2.1 GLP1R, but not GCGR, is expressed in human and mouse circumventricular organs

**Figure 1:**
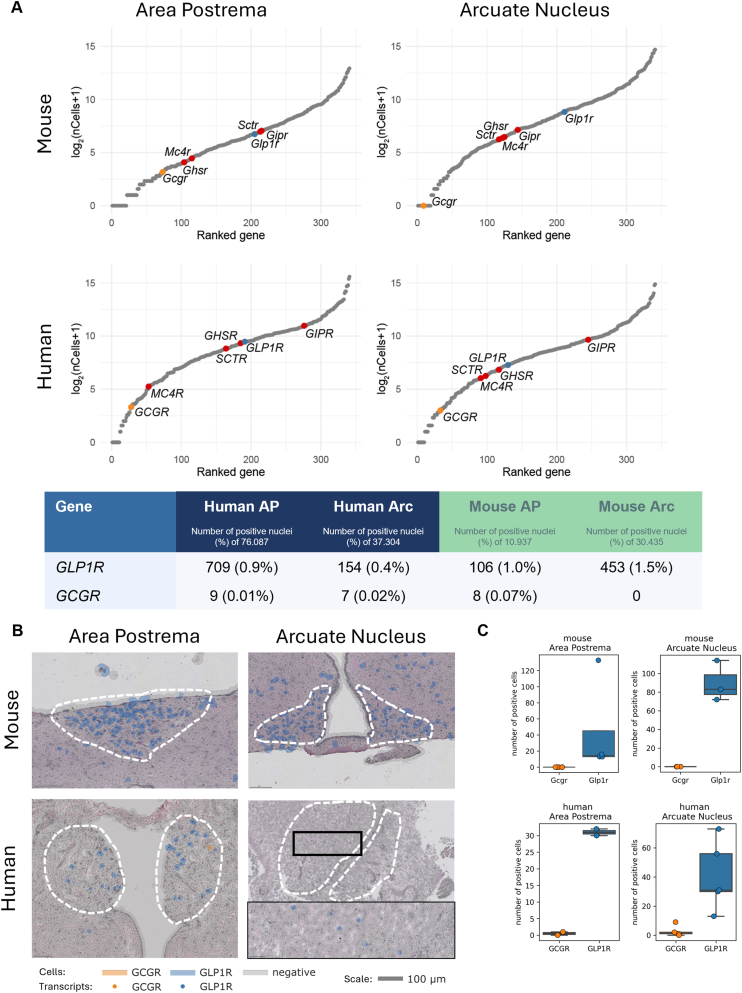
**Expression of *GLP-1R* and *GCGR* in human and mouse circumventricular organs.** Expression of GPCR-encoding genes in single nucleus RNA-seq data **(A)**. Within each panel, individual GPCR-encoding genes are represented by grey points and ranked according to the number of nuclei with non-zero counts (i.e., the total number of nuclei in which the respective genes were detected). *GLP1R* is highlighted in blue, *GCGR* in orange, other selected GPCRs related to metabolism in red. The table depicts the absolute and relative (%) number of nuclei in which the respective genes were detected in the respective datasets (6 donors respectively for human and mouse Arc and AP). Representative spatial gene expression analysis for *GCGR* and *GLP1R* via multiplex in-situ hybridization in the human and mouse AP (left) and the Arc (right) Each dot represents one individual transcript **(B)**. Number of *GLP1R* and *GCGR* positive cells in the areas of interest (mouse AP n = 4, mouse Arc n = 3, human AP n = 2, human Arc n = 5). Data are shown as mean ± SD. **(C)**. (For interpretation of the references to color in this figure legend, the reader is referred to the Web version of this article.)

To understand the site of action of survodutide in the brain, we first assessed expression of the target receptor genes *GCGR* and *GLP1R* in the CNS. We generated snRNA-seq data from micropunched human and mouse ARH and AP. We ranked G-protein coupled receptor-expressing genes in each dataset by the number of nuclei with non-zero counts (Figure 1A). Within these datasets, *GLP1R* was detected in the human and the mouse AP and ARC, respectively (between 0.4 and 1.5% of nuclei analyzed). In contrast, *GCGR* was virtually undetectable in the human and mouse ARC (human 0.02%, mouse 0%) and AP (human 0.01%; mouse 0.07%; Figure 1A). In addition, glucose-dependent insulinotropic polypeptide receptor (GIPR), secretin receptor (SCTR), melanocortin 4 receptor (MC4R), and growth hormone secretagogue receptor (GHSR) were ranked for comparison. We also evaluated *GL1PR* and *GCGR* expression in published HypoMap snRNA-seq datasets [20,22], focusing on predicted ARH neurons. In these data, GCGR transcripts were effectively absent, whereas GLP1R expression exhibited moderately higher prevalence relative to our own dataset.

(Supplementary Table 2). Furthermore, given the broad impact of GLP1R/GIPR co-agonists, we also assessed *GLP1R*/*GIPR* coexpression in our data. We observed almost completely mutually exclusive expression of these receptors in both ARH and AP (Supplementary Fig. 4).

In parallel to the analysis of single nuclei data, we generated spatial gene expression data via the Xenium in-situ technology (Figure 1B). Using this technology, we confirmed the findings for *GCGR* and *GLP1R* in transcript positive cells (Figure 1C) when compared to the single nuclei data. Additionally, other GPCRs (GIPR, GHSR, SCTR), known to modulate energy intake, were quantified in the same manner to provide context for the numbers of GLP1R- and GCGR-positive neurons (Supplementary Fig. 1A and B).

### 2.2 Survodutide-Cy7 activates the GLP-1R and GCGR in vitro and *in vivo*

**Figure 2:**
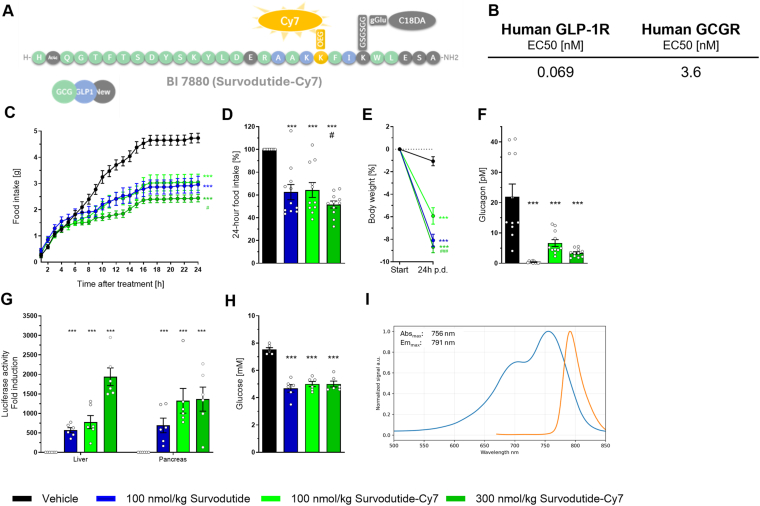
***In vitro* and *in vivo* characterization of survodutide-Cy7, a fluorescently labelled analog of survodutide.** Structure of survodutide-Cy7 **(A)**. *In vitro* potency (EC50 values) of survodutide-Cy7 on human GLP-1R and human GCGR in 0.5% human plasma **(B)**. 24-h food intake in g **(C),** % reduction in food intake over 24h **(D)**, and % reduction in body weight **(E)** after a single s.c. injection of survodutide or survodutide-Cy7 (n = 8/group). Plasma glucagon levels measured 24 h after injection **(F)**. Luciferase activity in the cAMP reporter CRE-Luc mouse (n = 6/group) i.v. treated with survodutide and survodutide-Cy7 **(G)**. Blood glucose levels 4h after compound injection **(H)**. Fluorescence spectra of survodutide-Cy7 **(I)** recorded on FluoroMax Plus. In vivo data are shown as mean ± SEM. Statistical analysis was done using one-way ANOVA followed by Dunnett's method for multiple comparisons versus vehicle with significance defined at ∗p < 0.05, ∗∗p < 0.01, ∗∗∗p < 0.001 and versus survodutide-Cy7 300 nmol/kg with significance defined at #p < 0.05 and ###p < 0.001. GLP-1R, glucagon-like peptide-1 receptor; GCGR, glucagon receptor.

To eventually be able to determine the central distribution of survodutide utilizing whole-brain 3D-imaging the fluorophore Cy7 was attached to the backbone of survodutide (Figure 2A). Prior to imagining studies, we characterized survodutide-Cy7, compared to survodutide alone, with respect to in vitro potency and alteration of caloric intake and energy balance. The in vitro potency of survodutide-Cy7 was determined in Cre-Luc HEK293 cells stably expressing human GLP-1R or GCGR as previously described [4]. Survodutide-Cy7 showed subnanomolar potency on the human GLP-1R (0.069 nM) which is similar to previously reported data for survodutide (EC50: 0.015 nM) with a lower potency on the human GCGR compared to survodutide (survodutide-Cy7 EC50: 3.6 nM vs survodutide EC50: 0.29 nM) (Figure 2B).

We tested the food intake lowering potency of survodutide-Cy7 in lean mice. A single dose of survodutide-Cy7 at 100 nmol/kg and 300 nmol/kg lowered food intake over 24h and body weight to the same extent as 100 nmol/kg survodutide (Figure 2C,D, E). After 24h, survodutide and survodutide-Cy7 significantly lowered endogenous plasma glucagon, demonstrating indirect target engagement on the GCGR (Figure 2F). The reduction in glucagon secretion upon stimulation with a GCGR agonist has been shown in preclinical and clinical studies for survodutide, which is likely a physiological feedback loop [4,[31], [32], [33]].

Using the CRE-Luc transgenic mouse model [34], a reporter line that allows the assessment of ligand activation of GPCRs *in vivo*, we confirmed that survodutide-Cy7 simultaneously engages the GLP-1R and GCGR *in vivo* at pharmacologically active doses was compared with survodutide [4]. Similar to survodutide, survodutide-Cy7 led to significant agonism of the GCGR and GLP-1R *in vivo* as seen by increased luciferase activity in liver and pancreas, respectively (Figure 2G) and blood glucose was significantly reduced in survodutide and survodutide-Cy7 treated animals (Figure 2H). Survodutide-Cy7 exhibited a characteristic near-infrared fluorescence profile, with a maximal absorbance at 756 nm and a corresponding emission peak at 791 nm, as demonstrated by the normalized excitation and emission spectra (Figure 2I).

### 2.3 Survodutide-Cy7 accesses circumventricular organs in mouse brain

**Figure 3:**
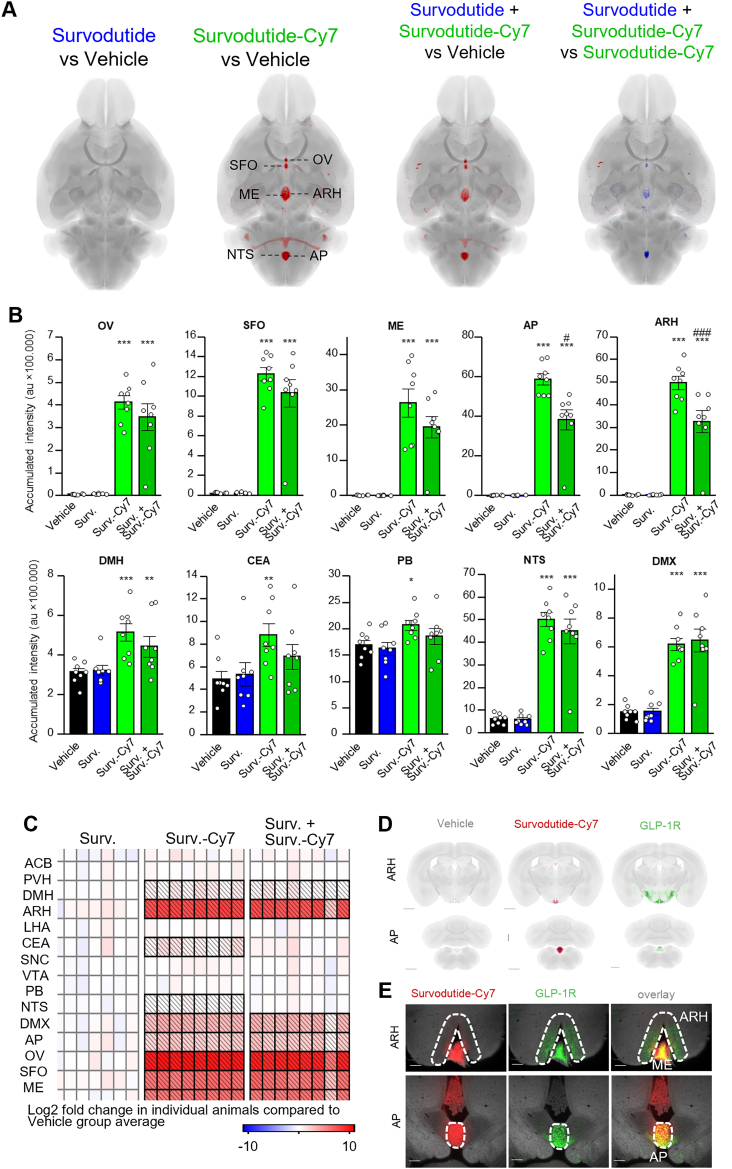
**Distribution of survodutide-Cy7 in the mouse brain**. 3D Whole-brain heatmaps (dorsal view) show vehicle-subtracted average signal intensity (red) of survodutide, survodutide-Cy7 and survodutide + survodutide-Cy7 competition experiment. On the right, group average fluorescence in mice pre-treated with non-fluorescent survodutide before fluorescent survodutide-Cy7 administration is compared to group dosed with survodutide-Cy7. Blue color indicates areas where fluorescence is lower in the pre-treated group **(A)**. Accumulated fluorescence intensity (arbitrary units, au) in selected brain regions. Standard error of the mean is shown. Bar charts demonstrate group average signal and spheres indicated individual animals. Statistical analysis was done using one-way ANOVA followed by Dunnett's method for multiple comparisons versus vehicle with significance defined at ∗∗∗p < 0.001. Survodutide + Survodutide-Cy7 group was additionally compared to Survodutide-Cy7 group, with #p < 0.05; ###p < 0.001, n = 8/group **(B)**. Signal intensity in every animal in each group compared to the mean intensity in the Vehicle treated group depicted as a heatmap. Areas that show statistically significant changes in signal intensity are highlighted in bold **(C)**. Optical coronal sections showing survodutide-Cy7 (red) distribution in arcuate hypothalamic nucleus (ARH) and area postrema (AP) and GLP-1R distribution is depicted in green. Scale bars, 1 mm **(D)**. Coronal sections showing distribution of survodutide-Cy7 and GLP1-R + neurons in arcuate hypothalamic nucleus (ARH) and area postrema (AP) after acute administration of the compound and anti-GLP1-R immunostaining. Tissue autofluorescence is shown in grey, signal from survodutide-Cy7 in red, and signal from GLP1-R in green. Scale bar 200 μm **(E)**. Brain region abbreviations: Nucleus accumbens (ACB), paraventricular hypothalamic nucleus (PVH), dorsomedial nucleus of the hypothalamus (DMH), arcuate hypothalamic nucleus (ARH), lateral hypothalamic area (LHA), central amygdalar nucleus (CEA), substantia nigra pars compacta (SNC), ventral tegmental area (VTA), parabrachial nucleus (PB), nucleus of the solitary tract (NTS), dorsal motor nucleus of the vagus nerve (DMX), area postrema (AP), vascular organ of the lamina terminalis (OV), subfornical organ (SFO), median eminence (ME). (For interpretation of the references to color in this figure legend, the reader is referred to the Web version of this article.)

To visualize distribution of survodutide-Cy7 within the central nervous systems, survodutide-Cy7 or vehicle control were injected i.v. acutely in lean mice with or without pre-treatment with survodutide, followed by tissue clearing and whole brain imaging using light sheet microscopy. In comparison to the vehicle group, mice dosed with survodutide-Cy7 showed strong fluorescent signal in the circumventricular organs (CVOs), including the vascular organ of the lamina terminalis (OV), subfornical organ (SFO), median eminence (ME), and AP (Figure 3A). Non-fluorescently tagged survodutide did not exhibit an increased signal in any of these regions (Figure 3B). The survodutide-Cy7 dosed groups showed increased fluorescence in comparison to Vehicle also in the ARH, dorsomedial nucleus of the hypothalamus (DMH), central amygdalar nucleus (CEA), parabrachial nucleus (PB), dorsal motor nucleus of the vagus nerve (DMX), and nucleus of the solitary tract (NTS) (Figure 3B). Overall, fluorescence accumulation was strongest in the ME/ARH and AP/NTS regions. Compared to the vehicle group, no significant fluorescence was detected in other brain regions associated with satiety and body weight regulation (ACB, PVH, lateral hypothalamic area (LHA), SNC, VTA) (Figure 3C). To demonstrate the specificity of survodutide-Cy7 in targeting receptors in particular brain regions, statistical comparison of survodutide-Cy7 dosed mice was carried out against mice pre-treated with non-fluorescent survodutide before survodutide-Cy7 administration (competition experiment). This analysis illustrated significantly weaker fluorescence in the AP and ARH in mice pre-treated with non-fluorescent survodutide, suggesting specific targeting of receptors in these regions (Figure 3A–B). To evaluate whether survodutide-Cy7 accesses GLP-1R positive areas, survodutide-Cy7 treated mouse brains were stained with GLP-1R antibody. The signal of survodutide-Cy7 in ARH and AP colocalizes with GLP-1R (Figure 3D,E, Supplementary Fig. 5) within the ARH but does not colocalize in the VMH.

### 2.4 Survodutide activates brain regions involved in controlling energy homeostasis

**Figure 4:**
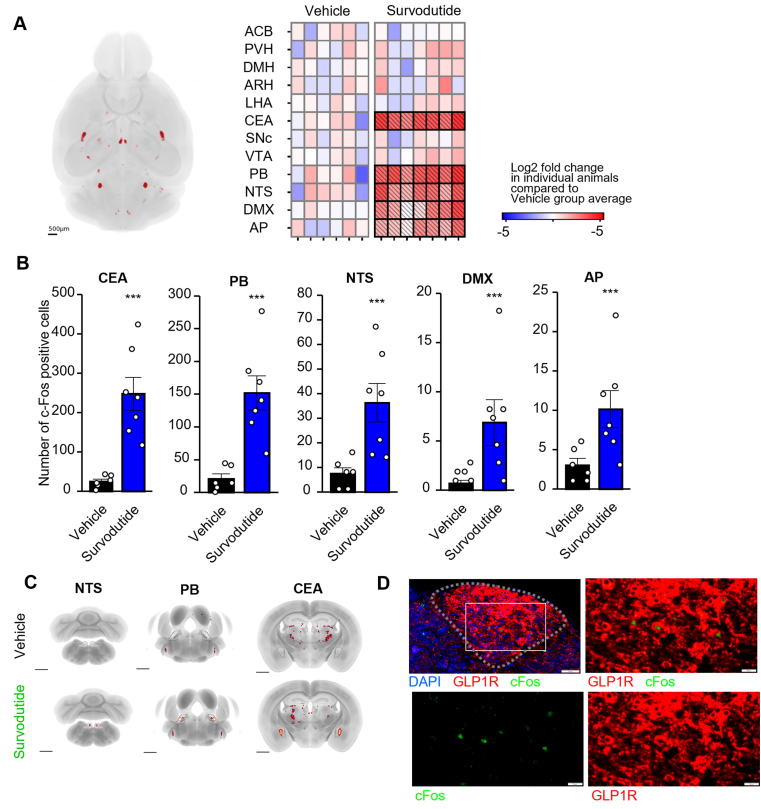
**Whole brain c-Fos expression in response to survodutide.** Group average c-Fos activity signature in response to s.c. survodutide dosing. Increased number of c-Fos positive cells compared to vehicle treated animals in red. Scale bar 1500 μm. Number of c-Fos positive cells in every animal in each group (n = 7) is compared to the mean value in the vehicle treated group (n = 6) and depicted as a heatmap. Areas that show statistically significant changes in c-Fos activity are highlighted in bold **(A).** Accumulated fluorescence intensity (arbitrary units, au) in selected brain regions. Standard error of the mean is shown. Bar charts demonstrate group average signal and spheres indicated individual animals. Statistical analysis was done using one-way ANOVA followed by Dunnett's method for multiple comparisons versus vehicle with significance defined at ∗∗∗p < 0.001. Brain region abbreviations: Nucleus accumbens (ACB), paraventricular hypothalamic nucleus (PVH), dorsomedial nucleus of the hypothalamus (DMH), arcuate hypothalamic nucleus (ARH), lateral hypothalamic area (LHA), central amygdalar nucleus (CEA), substantia nigra pars compacta (SNC), ventral tegmental area (VTA), parabrachial nucleus (PB), nucleus of the solitary tract (NTS), dorsal motor nucleus of the vagus nerve (DMX), area postrema (AP) **(B)**. Example 2D planes from mapped brains are shown at the level of the CEA, PB and NTS. Group average c-Fos signal is shown in glow color scale. Scale bars, 1 mm **(C).** Duplex immunohistochemistry in AP (dotted line) against c-Fos in green and GLP1R in red. White arrow indicates example of colocalization. Scale bar 20 μm **(D)**. (For interpretation of the references to color in this figure legend, the reader is referred to the Web version of this article.)

In a next step we assessed whole-brain activity signatures via expression of the immediate early gene c-Fos, which was quantified 4h after s.c. application of survodutide. In comparison to the vehicle treated group, acute dosing of survodutide resulted in significantly increased number of c-Fos labelled cells in brain regions associated with satiety and body weight regulations, i.e. in the CEA, PB, NTS, AP, and DMX (Figure 4A,B). Optical coronal sections (Figure 4C) from 3D imaged cleared brains further illustrate distinct c-Fos activation hotspots in NTS, PB and CEA. Within the AP (dotted line), c-Fos positive cells colocalized with GLP-1R positive neurons (Figure 4D).

### 2.5 LA-GCG does not activate neurons within circumventricular organs and does not lower body weight in diet-induced obese mice solely via reduction in food intake

**Figure 5:**
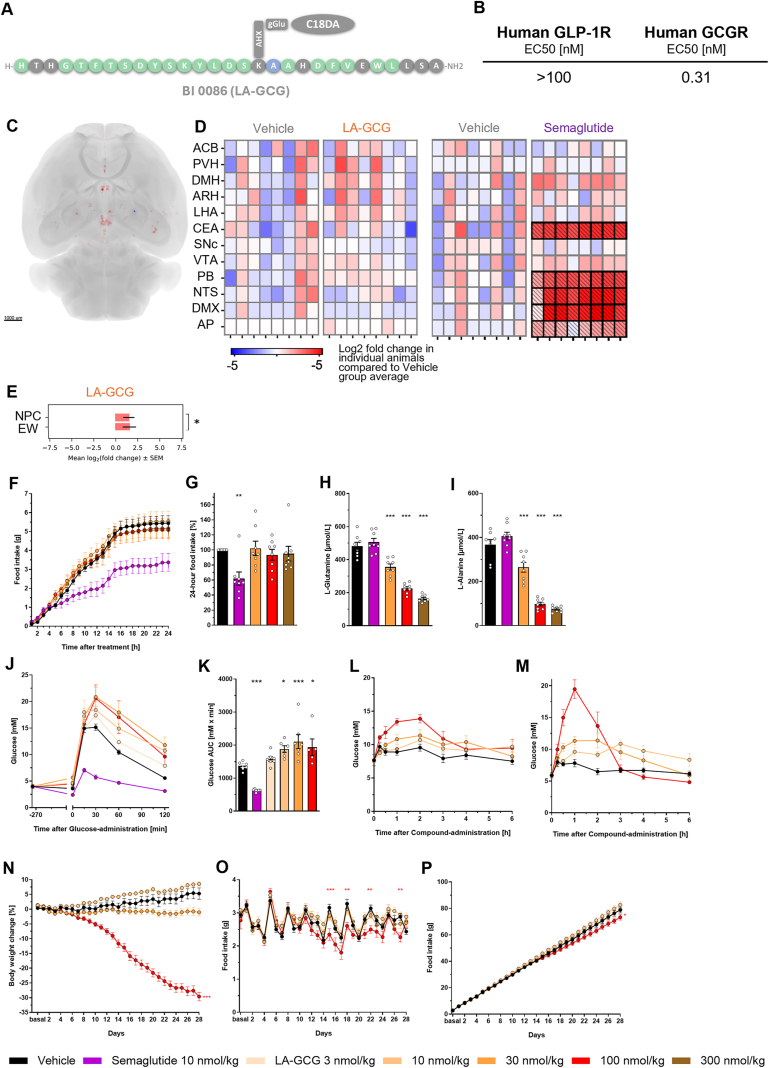
**Pharmacological characterization of LA-GCG and cFos response to LA-GCG administration.** Structure of LA-GCG **(A)**. *In vitro* potency (EC50 values) of LA-GCG on human GLP-1R and human GCGR in 0.5% human plasma **(B)**. Brain regions where the number of c-Fos positive cells is increased in response to s.c. LA-GCG in comparison to the vehicle group are shown in red and brain regions with reduced c-Fos labelled cell count compared to vehicle are shown in blue. Scale bar 1000 μm **(C)**. The number of c-Fos positive cells in every animal in each group (n = 8) is compared to the mean value in the vehicle treated group and demonstrated in heatmap scale. Areas that show statistically significant changes in c-Fos activity are highlighted in bold **(D)**. Bar chart demonstrating brain regions that are regulated by LA-GCG, ranked by significance. ∗: P < 0.05 compared to Vehicle, NS: not significant **(E).** Food intake over 24h after a single s.c. injection of LA-GCG and semaglutide (n = 8/group) **(F).** Cumulative food intake over 24h **(G).** Plasma amino acids l-glutamine **(H)** and l-alanine **(I)** levels measured 24 h after injection. Glucose tolerance tested by intraperitoneal glucose administration 6h after dosing with LA-GCG compared to semaglutide **(J, K)**. Glucose excursion after injection with LA-GCG in fed **(L)** and fasted state **(M)**. Effect of LA-GCG on body weight lowering **(N)** and food intake **(O)** over 28 days in diet-induced obese mice. Data are shown as mean ± SEM. Statistical analysis was done using one-way ANOVA followed by Dunnett's method for multiple comparisons versus vehicle with significance defined at ∗p < 0.05, ∗∗p < 0.01, ∗∗∗p < 0.001. GLP-1R, glucagon-like peptide-1 receptor; GCGR, glucagon receptor. (For interpretation of the references to color in this figure legend, the reader is referred to the Web version of this article.)

To further understand if survodutide acts via GLP-1R or GCGR in the brain, we investigated the pharmacology of a long-acting GCGR agonist (LA-GCG, [4]). LA-GCG (BI 0086) is a lipidated C18-diacid containing peptide with potent GCGR agonistic activity (LA-GCG EC50: 0.46 nM) and a >100-fold selectivity versus the GLP-1R (LA-GCG EC50: >63 nM) (Figure 5A,B), which was similar to native glucagon (GCGR EC50: 0.047 nM; GLP-1R EC50: 13 nM as previously reported, Supplementary Table 1) with an approximately 200-fold selectivity in this assay format. In contrast, native GLP-1 only agonizes the GLP-1R (EC50: 0.016 nM vs EC50 GCGR: >1000 nM, Supplementary Table 1).

When quantifying the whole-brain activity signatures via expression of the immediate early gene c-Fos, we found that LA-GCG did not induce c-Fos in brain regions involved in controlling energy homeostasis (Figure 5C–D). In comparison to the vehicle dosed group, a single dose of LA-GCG only resulted in increased number of c-Fos labelled cells in two brain regions: the nucleus of the posterior commissure (NPC) and Edinger-Westphal nucleus (EW) (Figure 5E). Furthermore, we compared the c-Fos response of survodutide to the c-Fos response of semaglutide in the same setting (Figure 5D). Semaglutide activated similar brain regions as survodutide, i.e. in the CEA, PB, NTS, AP, and DMX (Supplementary Fig. 3).

In acute studies, LA-GCG at doses up to 300 nmol/kg did not lower food intake after single administration to lean mice (Figure 5F,G), whereas semaglutide showed pronounced food intake reduction. However, LA-GCG significantly decreased plasma levels of amino acids l-glutamine and l-alanine after 24h (Figure 5H,I respectively), demonstrating *in vivo* target engagement of GCGR. In addition, LA-GCG worsened intraperitoneal glucose tolerance compared to an improved glucose tolerance with semaglutide (Figure 5J,K). Injection of LA-GCG in fed (Figure 5L) and fasted (Figure 5M) mice led to an increase in glucose excursion. After subchronic dosing in diet-induced obese mice, LA-GCG up to 100 nmol/kg lowered body weight dose-dependently up to 34% compared to vehicle. Food intake was not significantly reduced in the 100 nmol/kg group during the first 15 days, but showed reductions on individual days thereafter (Figure 5N, O, P). At day 28, fat and lean mass were considerably reduced at 100 nmol/kg (Supplementary Fig. 2A and B). Glucose levels were transiently increased throughout subchronic dosing (Supplementary Fig. 2C). Liver cholesterol and plasma triglycerides (Supplementary Fig. 2E and F) were not affected. Liver triglycerides, plasma cholesterol, and plasma glucagon (Supplementary Figs. 2D, G, H) were significantly decreased at day 28, with plasma glucagon as a biomarker demonstrating target engagement on the GCGR in the liver.

## 3 Discussion

In summary, our data support the hypothesis that survodutide modulates neuronal activity via activation of GLP-1Rs, at least in part, through a direct action and/or penetrance into adjacent CNS nuclei via CVOs to ultimately lower food intake. To our knowledge, this is the first time it is shown via single nuclei sequencing that *GCGR* is barely expressed in peptide-accessible brain regions and that activation of the GCGR via a long-acting GCGR agonist acutely neither affects neuronal activity nor food intake.

The expression of *GLP1R* within the ARH and AP has been demonstrated in this as well as in other recent studies [[18], [19], [20], [21], [22],^35^]. In contrast, the expression of *GCGR* in rodent and human tissue in brain has not yet been assessed at the single cell level up to date. Reports on expression of GCGR in rat ARH, ventromedial hypothalamus [36] or mouse mediobasal hypothalamus [16] are based on immunohistochemistry or western blot analysis with a commercially available antibody. Older studies regarding localization of *GCGR* in rat tissue are based on whole brain mRNA quantification [6,14]. Using bulk RNA sequencing, the Human Protein Atlas did not identify transcripts of GCGR in CVOs [37]. Here we show for the first time that *GCGR* is not expressed at the single cell level in CVOs in human and mouse brain. Although it is challenging to prove the absence of *GCGR* expression using the techniques we describe in this manuscript (snRNA-seq and in situ hybridization), the results presented in the context of other GPCRs clearly show that *GCGR* expression is at the far lower end of the distribution.

Survodutide is a peptide engineered to achieve a long half-life by the addition of a fatty acid half-life extension group [4]. The addition of a lipidation might impact CNS and deep-brain penetration, restricting the direct access to target cell populations in the CVOs and adjacent CNS nuclei (as well as the neuronal processes with known projection collaterals into these CVO-proxy nuclei) [38]. Our data demonstrate that survodutide does not penetrate deep into the CNS (e.g. VMH) but is restricted to CVOs (e.g. ARH and AP) and adjacent structures. Beyond the ARH and AP, our whole-brain imaging revealed that survodutide-Cy7 also accesses other circumventricular organs, including the subfornical organ (SFO) and the organum vasculosum of the lamina terminalis (OVLT). These regions, together with the median preoptic nucleus (MnPO), are implicated in fluid balance and have been shown to contain GLP-1R-expressing neurons that influence thirst regulation [39]. While our transcriptomic analyses were limited to ARH and AP due to technical and tissue availability constraints, the observed compound distribution suggests that survodutide may engage additional CVOs beyond those primarily associated with energy homeostasis. Future studies should explore receptor expression and functional contributions of these nuclei to fully delineate the central mechanisms underlying survodutide's effects on energy balance. With regards to technical feasibility of the survodutide-Cy7 distribution study a more pronounced reduction in fluorophore labeling was speculated and formed the rationale for the experimental setup involving survodutide pretreatment. Despite applying equimolar doses of 100 nmol/kg for both peptides, multiple dosing of survodutide prior to survodutide-Cy7 administration resulted in the observed outcome. This can be partly explained by the high GLP-1R in vitro potencies for survodutide (0.015 nM) and survodutide-Cy7 (0.069 nM). However, the results are in line with similarly lipidated peptides like semaglutide and GIPR agonists [23,40] and their fluorophore analogues. Liraglutide, as well as semaglutide, exhibit limited deep brain access following peripheral administration and activate neurons in the hypothalamus and the hindbrain that are involved in food intake control [23,24].

As survodutide enters the circumventricular organs (CVOs), it triggers a neuronal activation pattern that is known to alter food intake through downstream projections, a mechanism common to GLP-1R agonists [23,[41], [42], [43]].

While our study provides a comprehensive transcriptomic characterization of GCGR and GLP-1R expression in human and mouse circumventricular organs, it is important to note that functional target engagement by survodutide was assessed exclusively in mouse models. The human data presented here are limited to receptor expression profiles derived from single nucleus RNA sequencing and spatial transcriptomics. As such, conclusions regarding the physiological relevance of these receptors in human CVOs remain speculative and warrant further investigation using functional approaches in human tissue. A further limitation of the present study is the lack of validation of survodutide-Cy7 in GLP1R and GCGR knockout mouse models.

Glucagon has been long suggested to act in the brain and to reduce food intake in both mice [36] and humans [[44], [45], [46]]. However, using a long-acting GCGR agonist we demonstrate for the first time that GCGR agonism neither affects neuronal activation nor food intake but leads to a body weight reduction attributed to an increase in energy expenditure upon subchronic treatment of DIO mice [47,48]. While increases in energy expenditure (e.g., exercise) or energy loss (e.g., SGLT2 inhibitors) have been associated with counterregulatory responses to maintain energy balance [49,50], future research on the role of GCGR agonism in controlling energy balance within liver [3] and kidney [51] is therefore warranted. Reports on reductions in food intake by native glucagon are based on experiments with acute infusion (humans) [30] or acute injections in animals (mice) [52] of native glucagon. The doses and respective plasma exposures in these studies might be sufficient to activate the GLP-1R. Although endogenous glucagon has been shown to activate the GLP-1R only at very high concentrations and is approximately 1000-fold less potent than GLP-1 at the endogenous receptor [4,28,29], these selectivity relationships apply strictly to native hormones under physiological conditions. Acylated dual agonists such as survodutide exhibit receptor pharmacology that diverges markedly from endogenous peptides due to their engineered structure, prolonged half-life, and the supraphysiological exposure levels achieved in both preclinical and clinical studies. Similar deviations from endogenous selectivity have been described for other dual-agonist molecules [53].

To explore a putative CNS contribution of GCGR agonism on food intake we used a long-acting GCGR agonist [4] that due to its pharmacokinetic profile and plasma exposure upon subcutaneous injection provides the advantage of selectively engaging the GCGR *in vivo*. With our experimental design we cannot exclude contributions of GCGRs outside the CNS to the long-term effects on body weight that might be attributed to the regulation of food intake. In our studies we did not observe an effect on food intake over 15 days in a DiO model. In addition, LY3324954, another potent selective GCGR agonist increased energy expenditure and dose-dependent weight loss in the absence of food intake reduction [54], supporting our results. Just recently the LA-GCG has been shown to control obesity-specific energy expenditure via persistent cAMP/PKA signaling in the liver without effects on food intake [55].

Given the limited penetration of survodutide in the deep brain and the absence of *GCGR* expression in CVOs, we conclude that survodutide likely acts via the GLP-1R in the brain to elicit its food intake lowering effects. Adding GCGR potency leads to an additional effect on energy expenditure and subsequent superior weight lowering effects [4] compared with selective GLP-1R agonism alone. These data support the dual GCGR/GLP-1R mode of action and survodutides current clinical investigation in people living with obesity or overweight with complications and with (NCT06066528) or without type 2 diabetes (NCT06066515).

## 4 Material and methods

### 4.1 Spatial gene expression analysis

Xenium experiments have been performed according to the manufacturer's protocol using the predesigned mouse/human brain panels supplemented by a set of custom genes where *GCGR* and *GLP1R* were included. Briefly, 5 μm sections of relevant tissues (mouse AP n = 4, mouse Arc n = 3, human AP n = 2, human Arc n = 5) have been mounted on Xenium slides and went through incubations on a thermocycler for probe hybridization, ligation, and amplification. The run has been processed with the instrument software version 1.8.2.1 (mouse)/1.9.2.0 (human) and analysis version 1.7.1.0 (mouse)/1.9.0.0 (human). All human biospecimens and associated personal related data provided by our partners have been collected under a valid consent document and according to applicable law (Netherlands Brain Bank, Netherlands Institute for Neuroscience, Meibergdreef 47, 1105 BA, Amsterdam). Brain regions were anatomically identified during sectioning and validated based on their expression profile after the Xenium run (*AGRP* for arcuate nucleus, *GLP1R*, *GIPR* for area postrema). Cells with n ≥ 2 transcript were counted as positive. Data are presented as mean ± SD.

### 4.2 Single nucleus RNA-seq

All human postmortem procedures and animal procedures were approved by the University of Pennsylvania Institutional Review Board and Institutional Animal Care and Use Committee, respectively.

#### 4.2.1 Human arcuate nucleus

Human arcuate nucleus samples were obtained from fresh frozen coronal brain sections containing the hypothalamus from 6 human donors (3 female, 3 male). Sections were transferred from −80 °C storage to −20 °C for 2 h prior to being placed on a prechilled one-inch-thick sterilized aluminum slab on dry ice. Using stereotaxic coordinates and neuroanatomical landmarks (Atlas of the Human Brain, 4th Edition; Mouse Brain in Stereotaxic Coordinates), bilateral arcuate nucleus micropunches were dissected and transferred to a 1.5 mL Eppendorf style tube on dry ice. A nuclei suspension was prepared using a single nucleus isolation kit according to manufacturer protocols (Invent Biotechnologies #BN-020). Nuclei suspension concentration was determined [56] and nuclei were loaded onto two independent 10x Genomics Chromium microfluidic controller captures at 20,000 nuclei per reaction using the 3′ gene expression assay (v3.1) according to manufacturer protocol and like our prior description [57]. Sequencing libraries were constructed and sequenced on the Illumina NovaSeq 6000 per manufacturer's protocol and as we described [58]. Sequencing reads were demultiplexed and aligned to a previously detailed human reference transcriptome [59] using CellRanger v7.1. Count data from the two captures were integrated using Seurat v5.0.1. The data were clustered using principal components derived from genes with variable expression and SoupX [60] was used to correct for ambient RNA contamination. Low quality nuclei with ≤200 genes captured or ≥10% mitochondrial transcripts and putative doublets with ≥5000 genes captured were removed as standard quality control. Additional doublets were identified using scDblFinder [61] and removed. Finally, all clusters with abnormally low average UMI, mixed cell type markers, or identified as majority doublet were removed, resulting in a final dataset of 37,304 nuclei for human arcuate nucleus.

#### 4.2.2 Human area postrema

Single nucleus RNA sequencing libraries were generated as described above using AP micropunches from 8 human donors (5 female, 3 male). Count data from all samples were integrated using Seurat and clustering and quality control was performed as above. The final cleaned dataset contained 76,087 nuclei for human area postrema.

#### 4.2.3 Mouse arcuate nucleus and area postrema

Six Male C57BL/6J mice were purchased from the Jackson Laboratory and socially housed with ad libitum food and water for two weeks for acclimatization. Mice were euthanized via rapid decapitation, the brains were quickly removed and frozen in dry ice-cold hexane, and brains were stored at −80 °C. Two hours prior to microdissection, the fresh frozen brains were moved to a −20 °C cryostat for temperature equalization and then mounted onto a cryostat (Leica, CM3050S). Using stereotaxic coordinates and neuroanatomical landmarks (Paxinos, 5th Edition), bilateral micropunches of the arcuate nucleus and area postrema were collected and stored in 1.5 mL Eppendorf style tube on dry ice. Nuclei suspensions and single nuclei transcriptomics sequencing libraries were prepared as described above. Bioinformatics was conducted as described, with the data being aligned to a previously detailed mouse reference transcriptome [59]. The final cleaned dataset contained 30,435 nuclei for arcuate nucleus and 10,937 nuclei for mouse area postrema.

### 4.3 Peptide synthesis

Survodutide, LA-GCG, and semaglutide were synthesized as recently described [4]. The precursor of Cyanine7-labelled (Cy7) survodutide was synthesized by microwave-assisted SPPS using an Fmoc-strategy in dimethylformamide (DMF) on a polystyrene resin. N,N′-Diisopropylcarbodiimide (DIC) was used as coupling reagent together with ethyl(2Z)-2-cyano-2-(hydroxyimino)acetate (Oxyma) as base. Piperidine (10% in DMF) was used for deprotection. Synthesis until position 24 Dde-Lys(Fmoc)-OH was followed by Dde-Lys deprotection with hydrazine (5% in DMF) and assembly of the half-life extension group. Backbone synthesis was continued using Fmoc-L-Lys(Boc-AEEA)-OH (Combi-Blocks, San Diego, CA, USA) in position 21 and Fmoc-Lys(Dde)-OH in positions 20 and 12. Fmoc/Dde-protected precursor peptide was cleaved from resin using 95/2.5/2.5% (v/v) TFA/TIS/Wasser at 40 °C for 45 min, precipitated and purified by HPLC/MS. Cy7 labeling was carried out with Cy7-N-hydroxysuccinimide ester (Lumiprobe, Hannover, Germany) in DMSO using (N,N-diisopropylethylamine) DIPEA as base for 2 at RT. Deprotection with hydrazine was followed by neutralization with 5% TFA and purification by HPLC/MS. Lyophilization resulted in survodutide-Cy7. Purity and mass of survodutide-Cy7 was confirmed by HPLC/MS (MW_iso_ = 4918.57 Da, M + [1/3M + H] M_exp_ = 1640.53 Da, M_obs_ = 1640.52 Da).

### 4.4 In vitro functional receptor potencies

To characterize the impact of plasma protein binding for fatty-acid protracted peptides, the cAMP responsive element (CRE)-induced luciferase activity assay was applied, and receptor potencies were determined in 0.5% human plasma [4]. Human HEK293 CRE-luc2P cells (Promega, Madison, WI, USA) expressing recombinant GLP-1R and GCGR were cultivated in Dulbecco's Modified Eagle Medium (with high glucose/l-glutamine) supplemented with 10% fetal bovine serum, 50 μg/mL hygromycin, and 400 μg/mL Geneticin™. For the assays, cells were resuspended in KRBH supplemented either with 0.5 human plasma. Cells were treated for 4 h at 37 °C with the different peptides (all n = 3; all tested peptides were produced in house, handled as 1 mM stock solution in dimethyl sulfoxide, and tested at a final concentration range between 0.2 pM and 1 μM). In vitro potency was assessed using the Bright-Glo™ Luciferase Assay System (Promega), measuring the production of cAMP through a CRE-controlled luciferase. The potency (EC50) was calculated for each of the peptides.

### 4.5 Inhibition of acute food intake in NMRI outbred mice

Experimental protocols concerning the use of laboratory animals were reviewed by a federal ethics committee and approved by governmental authorities. All animal experiments comply with the ARRIVE guidelines and were carried out in accordance with the EU Directive 2010/63/EU for animal experiments.

Unless otherwise stated, the vehicle used throughout the studies was composed of 50 mM phosphate buffer pH 7.0 and 5% mannitol. All agent dosing was carried out at a volume of 5 mL/kg.

Three-week-old, male, lean NMRI outbred mice (wild-type [WT] were obtained from Janvier Labs (Le Genest-Saint-Isle, France) and were group-housed four mice per cage on a 12 h/12 h dark/light cycle (lights off at 15:00). The room temperature was controlled at 21 °C ± 1 °C, with 60% ± 20% humidity. Animals had ad libitum access to regular rodent chow (KLIBA NAFAG 3430; Granovit AG, Kaiseraugst, Switzerland) and tap water. Six-week-old male mice were randomized into treatment groups based on food intake and bodyweight (n = 8–10 per group). Mice were fasted for 6 h before randomization and SC dosing of survodutide, survodutide-Cy7, LA-GCG, semaglutide or vehicle 1 h before the dark phase. Food intake was measured for 24 h using a fully automated Herdsman-2 food-intake monitoring system (MBRose, Faaborg, Denmark). Blood was drawn at 24 h post dosing, and glucagon and amino acid levels were measured as recently described [4].

### 4.6 Luciferase activity in cAMP response element-luciferase transgenic mice

Male cAMP response element (CRE)-luciferase (Luc) transgenic mice (FvB/NTac-Tg [CRE/Tk-luc]187Hgp; Taconic Biosciences, Inc., Rensselaer, NY, USA) were obtained at 12 weeks old. Animals had ad libitum access to regular rodent chow (KLIBA NAFAG 3430; Granovit AG, Kaiseraugst, Switzerland) and tap water. Mice were dosed according to individual bodyweight with IV vehicle, survodutide or survodutide-Cy7. After 4 h, blood glucose was measured, and liver and pancreas were harvested and homogenized. Luciferase activity was measured using the luciferase assay system (Promega) [38].

### 4.7 Acute glucose tolerance tests and glucose excursion in lean mice

The effect of LA-GCG on acute glucose tolerance was assessed by intraperitoneal glucose tolerance test. Male lean C57BL6/J mice (Janvier Labs), 10–12 weeks old, were randomized (n = 6 per group) and fasted for 12 h prior to study initiation. Mice were administered SC LA-GCG or semaglutide at −4 h. Baseline blood glucose was measured at −4.5h, and then an intraperitoneal bolus of 2 g/kg glucose was applied at 0 h. Blood glucose was measured at 0, 15, 30, 60, and 120 min. Area under the curve (AUC) was calculated from glucose concentrations measured between 0 and 120 min. To determine glucose excursion after injection of LA-GCG, glucose was measured in fed or 6h fasted mice and immediately after LA-GCG injection at 15 min, 30 min, 1h, 2h, 3h, 4h, and 6h. In all experiments, glucose was measured in whole blood using a commercially available glucometer (GlucoSmart Swing) with test strips (MSP bodmann GmbH, Bobingen, Germany).

### 4.8 Subchronic, repeated dosing study in diet-induced obese mice

Male C57BL6/J mice pre-fed with a 60% high-fat diet (HFD) were obtained from The Jackson Laboratories at >16 weeks of age. Upon arrival, mice were single housed to obtain accurate and individual food intake measurements for each animal. During the entire study, animals had ad libitum access to food (HFD D12492; Research Diets, Inc., New Brunswick, NJ, USA) and tap water. Before the start of the study, animals were randomized based on their bodyweight measured 1 week prior to the start of treatment (n = 11 per group). At study start, the age of the mice was 22 weeks. Mice were administered chronic repeated subcutaneous (SC) dosing of vehicle or LA-GCG at 10, 30, or 100 nmol/kg daily for 28 days, with body weight and food intake measured every day. Glucose levels were determined before compound injection in the morning at 9 am at day 1, 2, 9, 16, and 23. At Day 28, EchoMRI 4in1-900 (EchoMRI, Houston, TX, USA) was carried out to determine lean and fat mass. At study termination at day 28, 4h post dosing blood was drawn to determine multiple biochemistry markers. Plasma triglycerides, cholesterol, and glucagon were measured. The liver was homogenized, and concentrations of triglycerides and cholesterol were measured. Plasma and liver triglycerides and cholesterol were measured using an automated analyzer (Cobas Integra 400; Roche). Plasma glucagon was measured using an enzyme-linked immunosorbent assay and colorimetric kits (MSD Metabolic Panel; Meso Scale Diagnostics, Rockville, MD, USA).

### 4.9 Survodutide-Cy7 distribution study

A total of 32 male, lean 9-week-old C57BL6/J mice (4 groups of n = 8) were tested for *in vivo* distribution of fluorescently labelled survodutide (survodutide-Cy7). 32 mice were stratified into four groups based on body weight. Group 1 was dosed SC on Day 1 with the vehicle and terminated after 1 h. Group 2 was dosed SC with 100 nmol/kg survodutide twice on day 1 and twice on day 2, followed by termination 1 h after the last dose. Group 3 was dosed IV on day 2 with 100 nmol/kg survodutide-Cy7 and terminated after 1 h. Animals in Group 4 were pre-treated with 100 nmol/kg survodutide three times (twice on day 1 and once on day 2), before they were injected with 100 nmol/kg survodutide-Cy7 on day 2 and terminated 1 h later. At termination, mice were intracardially perfused with heparinized PBS followed by perfusion fixation with 10% NBF (10% neutral buffered formalin). The isolated intact brains were immersion fixed in NBF overnight. The following day the samples are washed 3 × 30 min in PBS with shaking and dehydrated in methanol/H2O series: 20%, 40%, 60%, 80% and 100% for 1 h each at room temperature and incubated in 100% methanol overnight. Samples were next rehydrated in decreasing concentrations of methanol (80%, 60%, 40% and 20%) in PBS + 0.2% Triton X-100 for 1 h each at room temperature, followed by 1 h and overnight (room temperature) incubation in PBS + 0.2% Triton X-100. The next day, samples were dehydrated in methanol/H2O series: 20%, 40%, 60%, 80% and 100% for 1 h each at room temperature. Then, samples were incubated in 100% methanol overnight. The next day, samples were incubated overnight in 66% dichloromethane (DCM)/33% methanol at room temperature with shaking. Followingly, the samples are incubated in 100% DCM with shaking 2 × 1 h to remove traces of methanol. The samples were finally transferred to ECi and stored in closed glass vials in the dark. Light sheet microscopy was carried out as indicated above (imaging autofluorescence and BI-Cy7 signals only) and image analysis for compound biodistribution was carried out as previously described [38].

Brains (n = 2) from additional compound-dosed mice were immunostained with antibody against GLP-1R and cleared according to iDISCO protocol [62], with some modifications. The animals were at termination intracardially perfused with heparinized phosphate buffered saline (PBS) followed by cold (4 °C) glyoxal perfusion (80 ml glyoxal stock solution, Sigma–Aldrich #128465, diluted in 700 ml water, 20 ml ethanol, 7.5 ml acetic acid; adjusted to pH5). After perfusion fixation, the brains were carefully dissected, and immersion fixed in cold glyoxal overnight. The samples were next day washed 3 × 30 min in PBS with shaking. Samples from PBS were transferred to room temperature and dehydrated in methanol/H2O gradient (pH 9 adjusted): 20%, 40%, 60%, 80% and 100% methanol, each step 1 h (room temperature). They were washed in 100% methanol for 1 h and incubated overnight in 66%DCM (Dichloromethane)/33% methanol at room temperature. The next day the samples were washed twice in 100% methanol for 30 min. The samples were subsequently rehydrated in methanol/PBS series (pH 9 adjusted): 80%, 60%, 40%, 20%, with 0.2% Triton X-100, 1 h each at room temperature and washed in PBS (pH 9 adjusted) with 0.2% Triton X-100 (PTx.2) for 2 × 1 h at room temperature. Samples were incubated in permeabilization solution (pH 9 adjusted) at 37 °C for 3 days. Blocking (pH 9 adjusted) was carried out in blocking solution at 37 °C for 2 days. The samples were incubated with primary antibody (rabbit anti- GLP-1R, Abcam, ab218532) in PTwH/5%DMSO/3% donkey serum at 37 °C for 7 days. The specificity of the Abcam ab218532 antibody has been characterized previously [63]. Next, they are washed in PTwH (pH 9 adjusted) for 1 × 10 min, 1 × 20 min, 1 × 30 min, 1 × 1 h, 1 × 2 h and 1 × 2 days. Samples were incubated with secondary antibody (donkey anti-rabbit Cy5, Jackson Immunoresearch, 711-175-152) in PTwH/3% donkey serum at 37 °C for 7 days, followed by washes in PTwH (pH 9 adjusted): 1 × 10 min, 1 × 20 min, 1 × 30 min, 1 × 1 h, 1 × 2 h and 1 × 3 days. Tissue was cleared in methanol/H2O series (pH 9 adjusted): 20%, 40%, 60%, 80% and 100% for 1 h each at room temperature. Samples were incubated in 100% methanol overnight and next day for 3 h (with shaking) in 66%DCM (Dichloromethane)/33% methanol at room temperature and in 100% DCM 15 min twice (with shaking) to remove traces of methanol. The samples were finally transferred to ethyl cinnamate (Eci), stored in closed glass vials in dark. Brains were imaged using Lavision light-sheet ultramicroscope II (Miltenyi Biotec GmbH, Bergisch Gladbach, Germany) with Zyla 4.2PCL10 sCMOS camera (Andor Technology, Belfast, UK), SuperK EXTREME supercontinuum white-light laser EXR-15 (NKT Photonics, Birkerød, Denmark) and MV PLAPO 2 × C (Olympus, Tokyo, Japan) objective. Brains were mounted to a silicone sample holder (with ventral side up) and imaged in an ECi filled chamber. ImSpector microscope controller software (v7) was used (Miltenyi Biotec GmbH, Bergisch Gladbach, Germany). Images were acquired at 1.26× total magnification using dual-sided illumination and 9 horizontal focusing steps. Brains were imaged in autofluorescence (560 ± 40 nm excitation, 650 ± 50 nm emission), BI-Cy7 compound fluorescence (785 ± 25 nm excitation and 845 ± 55 nm emission) and antibody staining (630 ± 15 nm excitation and 680 ± 15 nm emission) wavelengths. Data was visualized using Imaris software 9.2 (Oxford instruments, Abington, UK).

### 4.10 c-Fos activation study with survodutide, LA-GCG and semaglutide

Diet-induced obese male (DIO) mice were treated with either Vehicle (n = 8) or survodutide (30 nmol/kg, n = 8), LA-GCG (100 nmol/kg, n = 8), or semaglutide (100 nmol/kg, n = 8). Animals were perfused 4 h after dosing and brains were dissected. Brain samples were labelled for c-Fos and cleared according to iDISCO + protocol as described previously [62,64]. Subsequently, the samples were 3D imaged using light sheet fluorescence microscopy as detailed above. Image analysis of the brain volumes included atlas mapping, detection, and quantification of c-Fos + cells per brain region, and region-wise statistical analysis for comparing the effect of treatment with survodutide to vehicle controls according to previously published method [64]. Furthermore, cleared and scanned brains were subsequently analysed for GLP1R immunoreactivity on paraffin embedded sections. Four cleared brains were washed 3 × 1h and overnight in 100% methanol. Samples were rehydrated in 99%, 96%, 70% ethanol for 1 h each and transferred to embedding cassettes and paraffin infiltrated overnight. 4 μm FFPE sections from the parabrachial nucleus and area postrema were cut. Tissue was deparaffinized (15 min in xylene, 99% ethanol for 2 × 3 min, 96% ethanol for 2 min, 70% ethanol for 2 min, rinsing in water for 5 min). Antigen retrieval was carried out using citric acid (pH6) for 10 min at 98 °C (KOS multifunctional tissue processor, Milestone). Peroxidase was blocked using 1% H_2_O_2_ in Tris-buffered saline (TBS) for 10 min, followed by 2 × 3 min washes in TBS-T (0.05% Tween 20). Slides were incubated in blocking buffer (TBS-T + 5% swine serum + 1% bovine serum albumin) for 20 min. Samples were incubated with anti-GLP1R antibody (Abcam ab218532, 0.25 ug/ml dilution) and anti-c-Fos antibody (Cell Signalling #2250, 1:100 dilution) diluted in blocking buffer for 1 h, followed by 3 × 3 min washes in TBS-T. Tyramine signal amplification (TSA)-Cy3 kit (Akoya Biosciences, NEL744B001KT) was used for detecting GLP1R signal and donkey anti-rabbit Cy5, (Jackson Immunoresearch, 711-175-152) for detecting c-Fos signal. Slides were scanned under 20x objective in an Olympus VS120 slide scanner.

### 4.11 Statistical analysis

Unless stated otherwise, data are presented as mean ± SEM and were compared using one-way ANOVA followed by two-sided Dunnett's method for multiple comparisons versus vehicle. Comparisons were considered significant at p < 0.05. Analyses were performed using GraphPad 9 statistical software (GraphPad Software).

Statistical analysis of the c-Fos + cells and accumulated signal intensity was performed by fitting a negative binomial generalized linear model (GLM) to the counts of 438 atlas-defined brain regions, for every study group. For each GLM, a two-sided Dunnett's test was performed. Statistical analysis was carried out using R packages MASS, multcomp, lmtest and car [[65], [66], [67], [68], [69]]. All significantly regulated regions were validated to ensure that the significance is not achieved due to overly influential datapoints (based on Cook's distance metric) and the signal is not originating from spillover of the neighboring regions.

## CRediT authorship contribution statement

**Tina Zimmermann:** Writing – review & editing, Writing – original draft, Visualization, Validation, Project administration, Investigation. **Katherin Bleymehl:** Writing – review & editing, Formal analysis, Data curation. **Peter Haebel:** Writing – review & editing, Resources, Methodology. **Johanna Perens:** Writing – review & editing, Visualization, Formal analysis, Data curation. **Urmas Roostalu:** Writing – review & editing, Visualization, Formal analysis, Data curation. **Jacob Hecksher-Sørensen:** Writing – review & editing, Visualization, Formal analysis, Data curation. **Jonas Doerr:** Writing – review & editing, Visualization, Formal analysis, Data curation. **Sebastian Jarosch:** Writing – review & editing, Visualization, Formal analysis, Data curation. **Daniel Lam:** Writing – review & editing, Visualization, Formal analysis. **Holger Klein:** Writing – review & editing, Resources, Conceptualization. **Anton Pekcec:** Writing – review & editing, Resources, Conceptualization. **Samar N. Chehimi:** Writing – review & editing, Investigation. **Richard C. Crist:** Writing – review & editing, Formal analysis, Data curation. **Benjamin C. Reiner:** Writing – review & editing, Resources, Formal analysis. **Matthew R. Hayes:** Writing – review & editing, Resources, Conceptualization. **Robert Augustin:** Writing – review & editing, Writing – original draft, Supervision, Investigation, Conceptualization.

## Funding

This research did not receive any specific grant from funding agencies in the public, commercial, or not-for-profit sectors.

## Declaration of competing interest

The authors meet criteria for authorship as recommended by the International Committee of Medical Journal Editors (ICMJE). The authors did not receive payment related to the development of the manuscript. Boehringer Ingelheim was given the opportunity to review the manuscript for medical and scientific accuracy as well as intellectual property considerations. The study was supported and funded by Boehringer Ingelheim. TZ, KB, PH, JD, SJ, DL, HK, AP and RA are employees of Boehringer Ingelheim. UR is employed at Gubra. At the time of the study JP and JHS have been employees at Gubra and are now employed at Vibraint. B.C.R. and M.R.H. receive research funding from Boehringer Ingelheim. M.R.H. also receives funding from Coronation Bio, Pfizer, Gila Therapeutics, and Eli Lilly & Co, and these funds were not used in support of these studies. B.C.R. receives in-kind support from Oxford Nanopore Technologies that is not related to this project.

## Data Availability

Data will be made available on request.
