# Supplementary material for: Survodutide acts through circumventricular organs in the brain and activates neuronal regions associated with appetite regulation
Source: Mol Metab. 2026 Feb 2;105:102326. doi: 10.1016/j.molmet.2026.102326 (PMC12925197; doi:10.1016/j.molmet.2026.102326)
Supplement: Multimedia component 1 [file mmc1.docx]

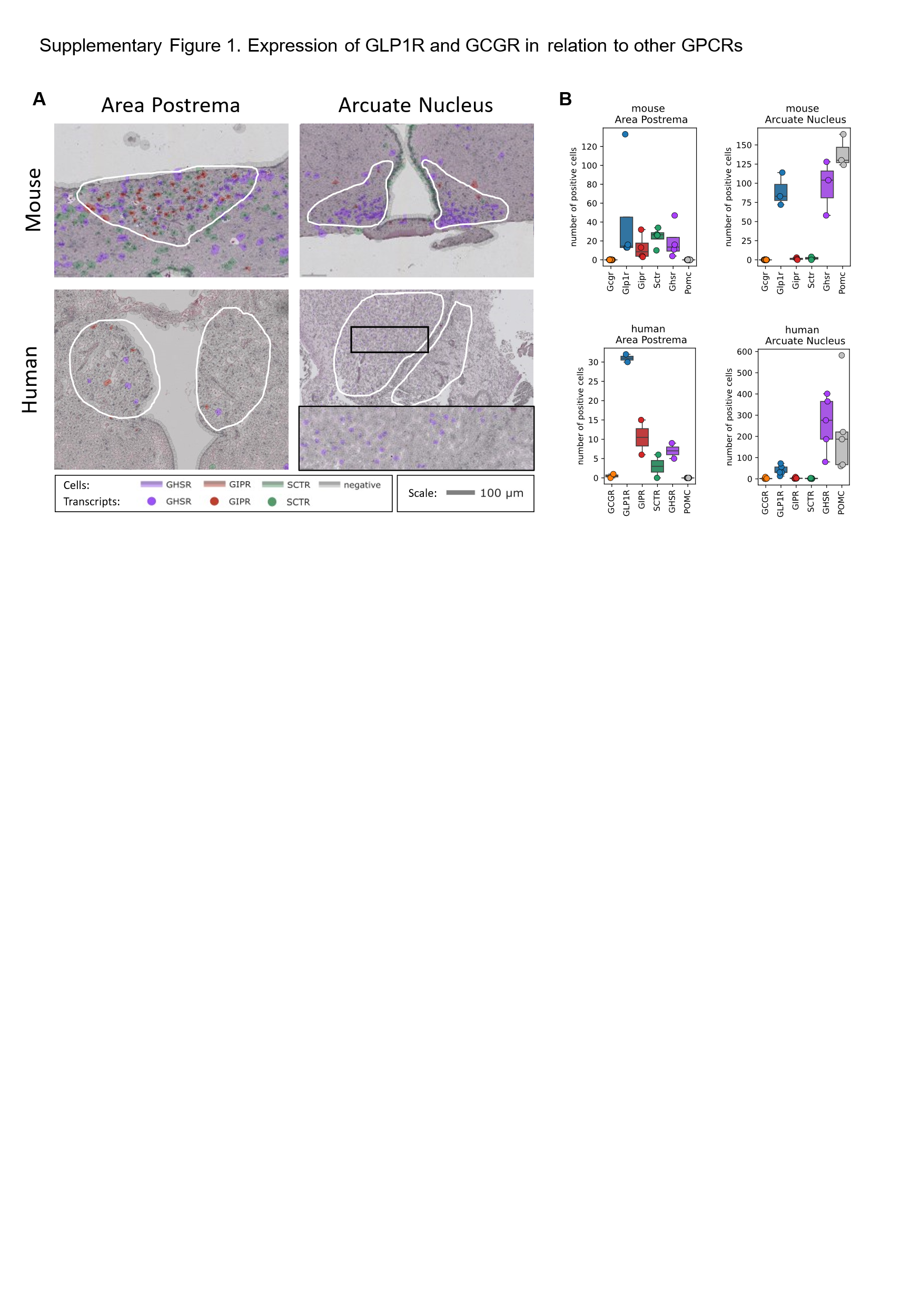


**Supplementary Figure 1. Expression of GLP1R and GCGR in relation to other GPCRs.** Representative spatial gene expression analysis for additional GPCRs in relation to *GLP1R* and *GCGR* (See Figure 1) via multiplex in-situ hybridization in the human and mouse area postrema (left) and the arcuate nucleus (right) **(A)**. Number of *GLP1R, GCGR, GIPR, SCTR, GHSR and POMC* positive cells in the areas of interest **(B)**. Cells with n≥2 transcript were counted as positive. Each dot represents one individual transcript.

**
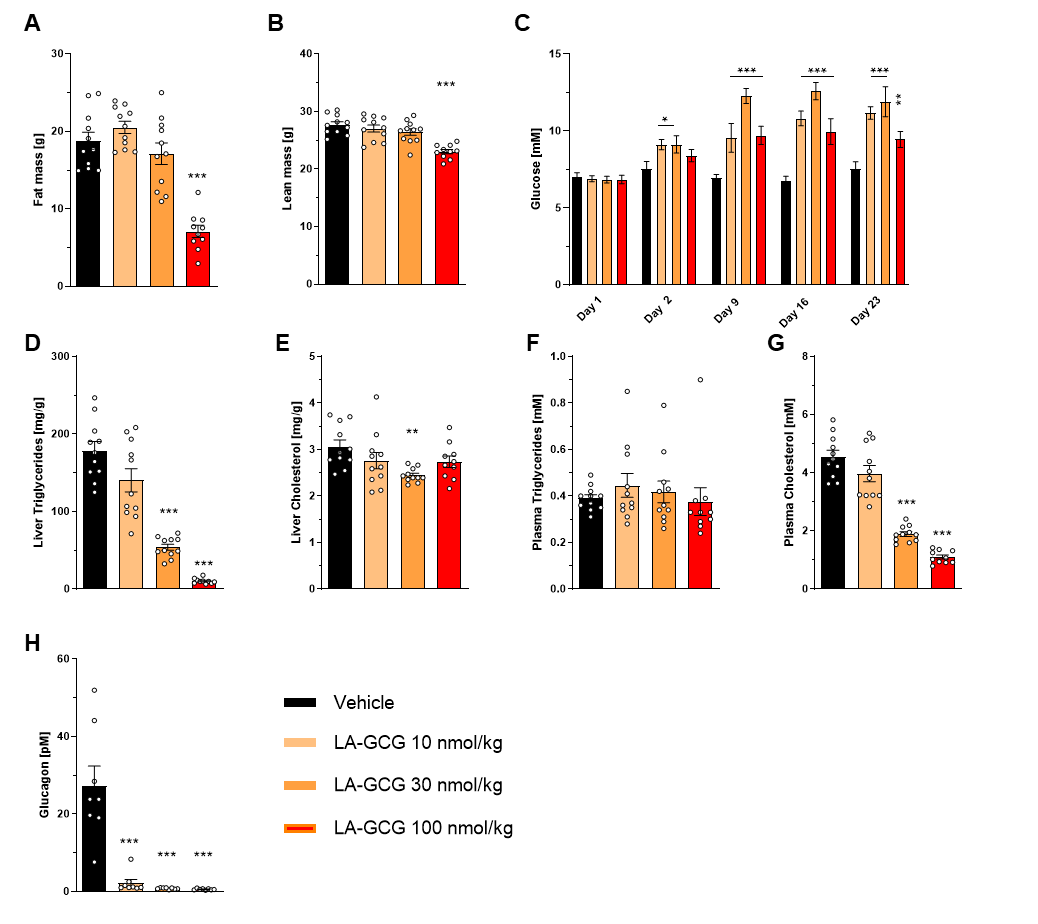
**

**Supplementary Figure 2. Effects of subchronic dosing of LA-GCG in DIO mice on metabolic parameters.** Day 28 fat mass (A) and lean mass (B) determined via EcoMRI. Blood glucose levels determined at day 1 before injection, day 2, day 9, day 16, and day 23 after injection with LA-GCG. Concentrations of liver triglycerides (D), liver cholesterol (E), plasma triglycerides (F), plasma cholesterol (G) and plasma glucagon (H) at day 28. Data are shown as mean ± SEM. Statistical analysis was done using one-way ANOVA followed by Dunnett’s method for multiple comparisons versus vehicle with significance defined at *p<0.05, **p<0.01, ***p<0.001.

**
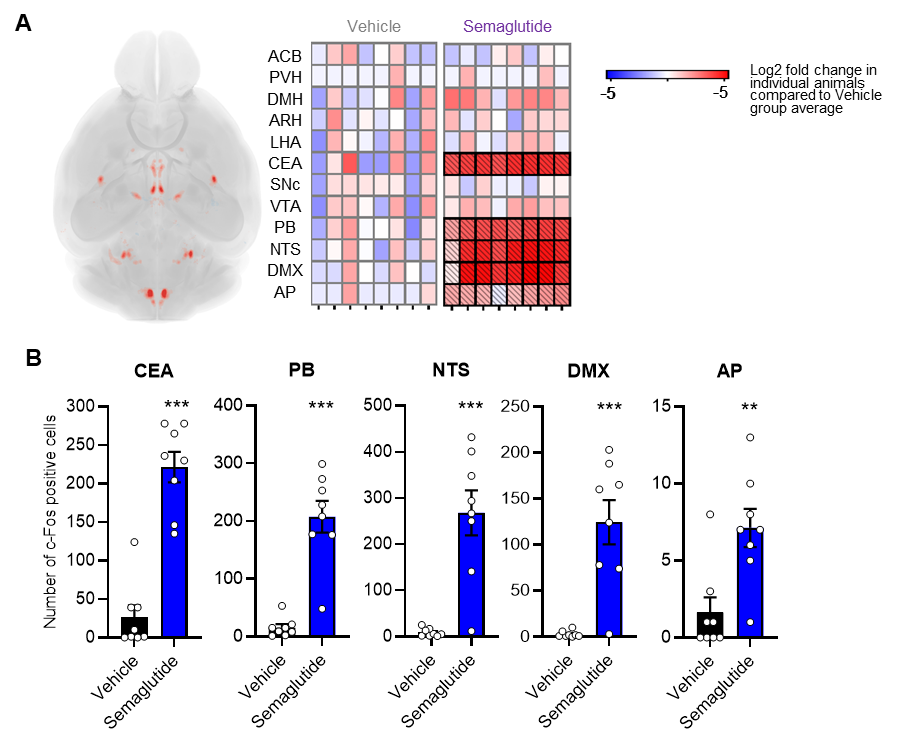
Supplementary Figure 3. cFos activation with GLP-1R agonist semaglutide.** Group average c-Fos activity signature in response to semaglutide dosing. Increased number of c-Fos positive cells compared to vehicle treated animals in red. Scale bar 1500 µm**.** Number of c-Fos positive cells in every animal in each group is compared to the mean value in the vehicle treated group and depicted as a heatmap. Areas that show statistically significant changes in c-Fos activity are highlighted in bold **(A).** Accumulated fluorescence intensity (arbitrary units, au) in selected brain regions. Standard error of the mean is shown. Bar charts demonstrate group average signal and spheres indicated individual animals. Statistical analysis was done using one-way ANOVA followed by Dunnett’s method for multiple comparisons versus vehicle with significance defined at ***p<0.001. Brain region abbreviations: Nucleus accumbens (ACB), paraventricular hypothalamic nucleus (PVH), dorsomedial nucleus of the hypothalamus (DMH), arcuate hypothalamic nucleus (ARH), lateral hypothalamic area (LHA), central amygdalar nucleus (CEA), substantia nigra pars compacta (SNC), ventral tegmental area (VTA), parabrachial nucleus (PB), nucleus of the solitary tract (NTS), dorsal motor nucleus of the vagus nerve (DMX), area postrema (AP) **(B)**.

**
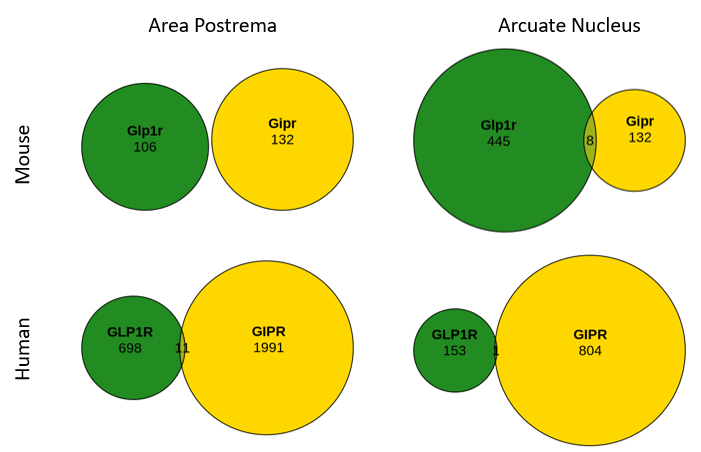
**

**Supplementary Figure 4. GLP1R/GIPR coexpression.** Number of nuclei in which *GLP1R*/*GIPR* were detected in each dataset, depicted as scaled Venn diagram to visualize coexpression.


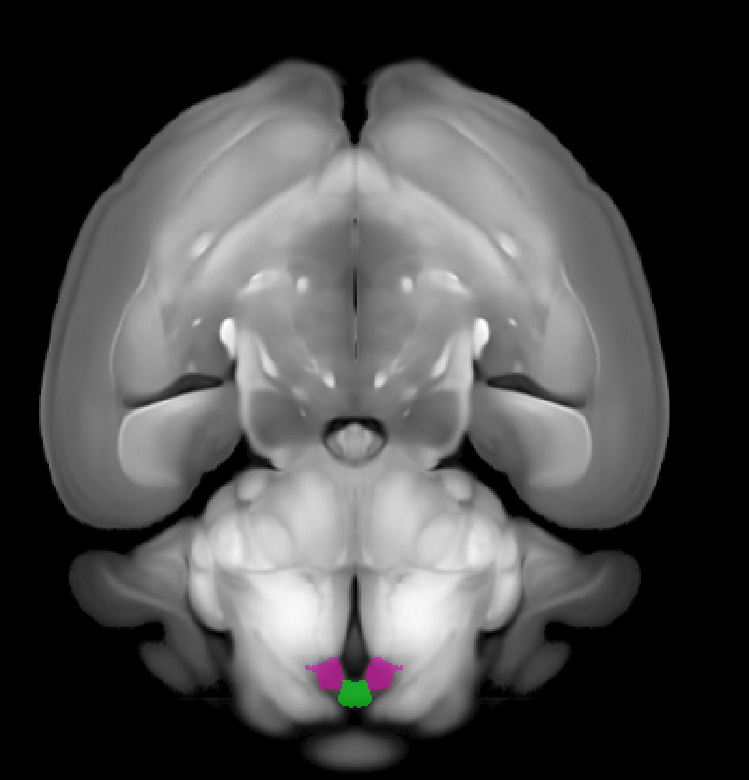

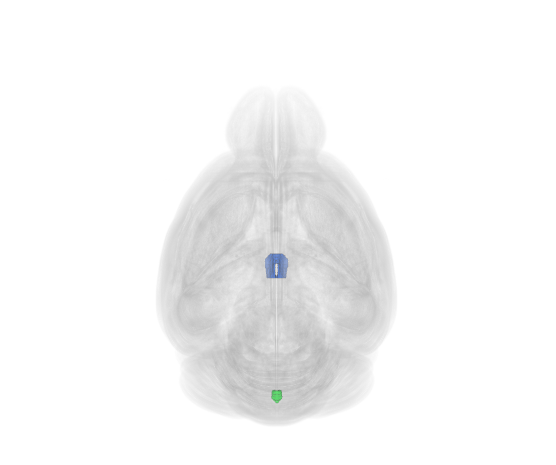


Bregma

**A**

**C**

**B**


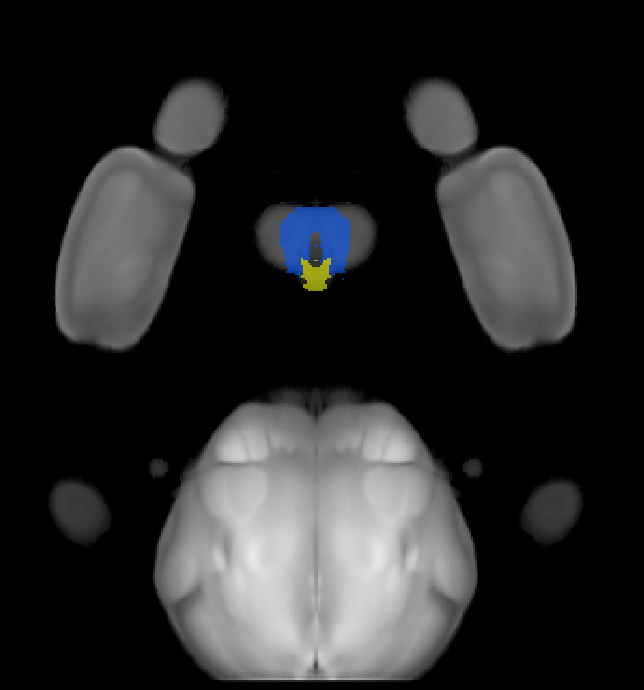


**Supplementary Figure 5. Image plane corresponding to Figure 3E. A.** Visualization of arcuate hypothalamic nucleus (ARH) in blue and median eminence (ME) in yellow on an optical section from light sheet mouse brain atlas. **B.** Visualization of area postrema (AP) region in green and nucleus of the solitary tract (NTS) in magenta on an optical section from mouse brain atlas. The images (A, B) represent horizontal planes, and red boxes indicate approximate positions corresponding to the images from representative samples in Figure 3E. **C.** Dorsal view on a 3-dimensional (3D) representation of a digital mouse brain atlas, illustrating ARH and AP positions and the position of Bregma.

**
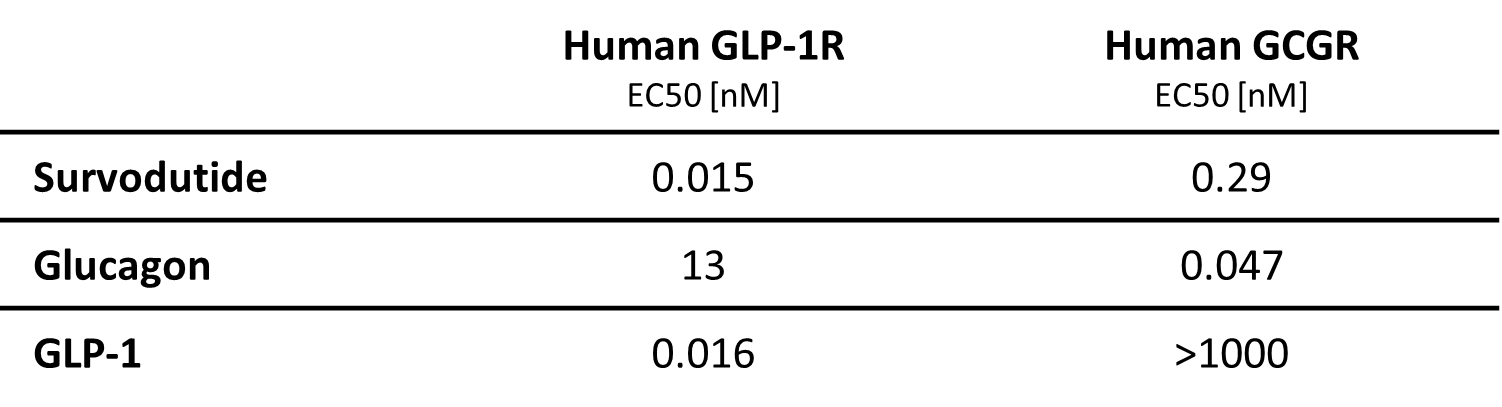
**

**Supplementary Table 1. In vitro potencies (EC50 values) of survodutide, GCG, and GLP-1 to activate the human GCGR or GLP-1R as previously reported [1].** The half-maximal effective concentration of survodutide, glucagon, and GLP-1 to agonize the human GCGR or the human GLP-1R were determined based on induction of luciferase activity in CRE-Luc cells recombinantly overexpressing the respective receptor in the presence of 0.5% human plasma.

**
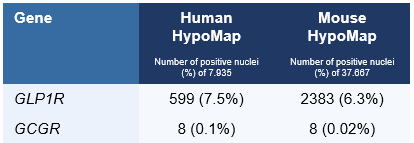
**

**Supplementary Table 2. *GLP1R/GCGR* expression in HypoMap datasets.** The number of nuclei in which expression of each receptor was detected is shown, as well as the percentage of total nuclei in each dataset. HypoMap data of the whole hypothalamus were subsetted to predicted ARH neurons (based on cluster-level spatial marker gene expression; methods implemented by HypoMap authors).
